# Validity and Reliability of the Glaucoma Knowledge Level Questionnaire

**DOI:** 10.4274/tjo.26576

**Published:** 2018-06-28

**Authors:** Zeynep Demirtaş, Gökçe Dağtekin, Muhammed Fatih Önsüz, Aziz Soysal, Nilgün Yıldırım, Selma Metintaş

**Affiliations:** 1 Eskişehir Osmangazi University Faculty of Medicine, Department of Public Health, Eskişehir, Turkey; 2 Eskişehir Osmangazi University Faculty of Medicine, Department of Ophthalmology, Eskişehir, Turkey

**Keywords:** Glaucoma, level of knowledge, scale development

## Abstract

**Objectives:**

The present study was conducted to develop an instrument for measuring adults’ glaucoma knowledge levels and to establish the instrument’s validity and reliability.

**Materials and Methods:**

The study group consisted of 811 persons aged 40-80 years who presented to primary health care institutions and did not have a glaucoma diagnosis. A 27-item questionnaire measuring level of glaucoma knowledge was created by the study team. Following expert consultation, it was structurally evaluated. The difficulty index and discrimination index were calculated for each item. Factor analysis was used to determine construct validity, Cronbach’s alpha internal consistency coefficient and item-total correlations were calculated to determine reliability. Confirmatory factor analysis was used to assess the extent to which the factor structure of the scale fit. We analysed correlation with the National Eye Health Education Program (NEHEP) Eye-Q scale in order to evaluate the validity of the scale.

**Results:**

The final glaucoma knowledge level questionnaire comprised 10 items in one dimension. The discrimination index and difficulty index ranged between 0.28 to 0.65 and 33 to 61%, respectively. According to factor analysis, the Kaiser-Meyer-Olkin score was 0.760 and Bartlett’s test indicated p<0.001. Confirmatory factor analysis showed acceptable scale fit and fit indices. Validity assessment revealed a positive correlation between the total score of the items of the NEHEP scale and glaucoma knowledge level questionnaire score (r=0.522; p<0.001). Scores were higher in participants who were aged 40-64, living in the city, had education level of high school or above and had previous eye examination or intraocular pressure measurement.

**Conclusion:**

The glaucoma knowledge level questionnaire has the distinction of being the first valid and reliable scale for assessing level of glaucoma knowledge in Turkey.

## Introduction

Glaucoma is an optic neuropathy characterized by loss of retinal ganglion cells, optic nerve atrophy and visual field loss.^1^ This global public health problem is the most common cause of blindness in the world after cataract.^2^ It is estimated that glaucoma affects more than 60 million people in the world and that this number will exceed 100 million by the year 2040. Because early glaucoma is usually asymptomatic, many people are unaware of the disease until the onset of vision loss.^3,4^ Early diagnosis can prevent glaucoma-related blindness and its adverse effects on quality of life.^5^ It is estimated that about 90% of glaucoma-related blindness can be prevented with early and appropriate treatment.^6^

Timely eye examinations and appropriate treatment are critical to reduce visual impairment and blindness caused by glaucoma. However, many people in developing countries do not have regular and timely eye examinations due to a lack of knowledge and awareness about glaucoma-related blindness.^6^ As glaucoma does not cause obvious symptoms such as pain, many people do not undergo screening for early diagnosis.^2^ Glaucoma awareness is especially low in rural areas and poor communities.^6^ Some authors have reported that awareness of glaucoma is insufficient even in Western societies.^7,8^ In addition to the early recognition of asymptomatic patients, the treatment of diagnosed patients is also an important link in controlling glaucoma. Therefore, knowledge and awareness about glaucoma must be increased among both the general population and glaucoma patients. Patient education has also been shown to improve treatment adherence.^1^

Although tools have been used in studies conducted on various populations to determine the level of knowledge regarding glaucoma and related risk factors, there are no valid and reliable tools for the Turkish population.

This study was conducted to develop a scale that assesses the knowledge level of Turkish adults about glaucoma and to ensure the validity and reliability of this scale.

## Materials and Methods

### Development and Content Validity of the Glaucoma Knowledge Level Questionnaire

First, we conducted a comprehensive literature review and identified items that measure glaucoma knowledge level. In the preparation of the glaucoma knowledge level questionnaire (GKLQ), 9 items from the glaucoma Eye-Q test^9^ developed by the National Eye Health Education Program (NEHEP) were translated into Turkish (one race-related item was excluded). A questionnaire of 27 items in total was created according to expert opinion determined through our review of the literature. Participants were asked to respond to each item as “correct”, “incorrect”, or “I do not know”. Eight of the items were reverse worded.

The appropriateness and comprehensibility of each item was evaluated by 8 specialists (1 ophthalmologist, 6 public health specialists and 1 ophthalmology nurse). The content validity ratio and content validity index of the scale were 0.82 and 0.87, respectively. The specialists were asked to rate each item as “important”, “useful but inadequate”, or “unnecessary”. The expert panel found one item (the reverse-worded “glaucoma is affected by a person’s diet”) unnecessary according to the content validity criteria and it was removed from the scale. A Turkish language expert (H.Ö.) evaluated the questionnaire and made necessary changes. A pilot study of the questionnaire was conducted with 10 participants, who were asked to add written comments and provide verbal feedback. All of the participants reported that the items were understandable. The Cronbach’s alpha coefficient for the pilot study was 0.47.

### Ethical Approval

Approval was obtained for the study from the Eskişehir Osmangazi University Ethics Committee (approval number 2016-9/5).

### Study Group and Procedure

The study was carried out in Eskişehir, Turkey between June and December 2016. Eskişehir is one of the developed provinces of Turkey and had a population of 844,842 in 2016. Eighty-seven percent of the population lives in the urban center and 13% live in rural areas.

The study included 811 participants aged 40-80 years and a random sampling method was used. The study group consisted of patients who were admitted throughout the duration of the study to primary health care institutions within the Eskişehir Osmangazi University Training and Research Region that was established by the Eskişehir Osmangazi University Faculty of Medicine for the purpose of conducting social research. Patients who were not diagnosed with glaucoma and were not taking any medication for glaucoma were included. Individuals who did not consent to participate in the research, who had communication problems and who did not respond to at least 90% of the questions in the questionnaire were not included in the study. Informed consent was obtained from all participants. 

In addition to the questions in the model scale, the participants filled out a questionnaire about sociodemographic characteristics such as age, education level, place of residence and income level. The questionnaire was completed in 10-15 minutes.

## Reliability Analysis

### Item Discrimination and Difficulty Indices

The item discrimination index and difficulty index were calculated for each item. To do this, the scores were first sorted in numerical order and divided into three groups. The difficulty index was calculated by dividing the number of people who answered the item correctly in the top 27% scoring group and the bottom 27% group by the total number of respondents in the top and bottom groups. If the item difficulty index is lower than 30%, the item is considered difficult. The item discrimination index indicates the degree to which an item discriminates between those who are knowledgeable and those who are not. The item discrimination index was calculated by subtracting the number of correct responders in the lower group from the number of correct responders in the upper group and dividing that figure by the total number of individuals in the lower or upper group (they are equal). Items with item discrimination index lower than 0.19 were considered very weak items that should be removed. Ultimately, 11 items with item difficulty index below 30% and item discrimination index below 0.19 were removed. These items were “Eye pain is common in glaucoma”, “Glaucoma occurs due to increased intraocular pressure”, “Loss of vision due to glaucoma can improve with treatment”, “A complete eye examination is done only by measuring intraocular pressure”, “There is more than one type of glaucoma”, “The treatment for glaucoma is usually surgery”, “Infections of the outer membrane of the eye can cause glaucoma”, “Blurred vision and headaches are common in glaucoma”, “Vision loss usually develops rapidly in glaucoma”, “Men are affected more by glaucoma than women” and “Doing light exercise such as walking lowers ocular pressure”.

### Internal Consistency (Reliability)

Cronbach’s alpha coefficient and item-total correlations were calculated to analyze the scale’s reliability. Items with an item-total correlation greater than 0.20 were considered reliable.^10^ Five items (“A person cannot understand that he/she has glaucoma”, “Individuals at high risk for glaucoma should have their pupils dilated for examination”, “Eye drops used for the treatment of glaucoma may cause ocular redness and burning”, “Individuals with distant or near vision problems are at risk for glaucoma” and “Overweight individuals are at risk for glaucoma”) had item-total correlations lower than 0.20 and were removed from the questionnaire. The reliability levels represented by the Cronbach’s alpha coefficient were as follows: 0.40 and below, unreliable; 0.40-0.60, low reliability, 0.60-0.80, very reliable and 0.80-1.00, highly reliable.^11^

### Factor Analysis

Factor analysis was used for construct validity. Factor analysis was done using principle components analysis (PCA) with varimax rotation. PCA is often used to reduce the number of items and determine pattern (in other words, the number and relationship of the main dimensions within the structure) when testing the psychometric properties of structured questionnaires. 

### Confirmatory Factor Analysis

Using Lisrel 8.8 software, confirmatory factor analysis (CFA) was done to assess the consistency of the scale’s factor structure. While exploratory factor analysis aims to find a factor or factors based on the relationships between variables, CFA tests a previously determined hypothesis about the relationship between variables.^12^ For confirmatory factor analysis, the most commonly used fit indices were calculated to assess the consistency of the model with the data. These indices included the Goodness of Fit Index (GFI), Adjusted Goodness of Fit Index (AGFI), Comparative Fit Index (CFI), Root Mean Square Error of Approximation (RMSEA) and Standardized Root Mean Square Residual (SRMR). Acceptable levels of fit for the indices were >0.90 for GFI, CFI and AGFI, <0.08 for RMSEA and SRMR.^13^

### Scoring

The final scale consisted of 10 items and 1 dimension. One of the items was reverse worded. Responses to the statements were scored as 2 if correct, 1 if “I don’t know” and 0 if incorrect. The reverse worded item was reverse coded to the other items. The scale had a maximum score of 20 and minimum score of 0.

### Validity

To assess the validity of the GKLQ, Spearman’s correlation analysis was used to compare total GKLQ scores with total scores of the items of the Eye-Q Test, a widely accepted scale developed by the NEHEP.

### Data Analysis

The data were analyzed using the IBM SPSS 15 software package. Descriptive statistics of the study group were reported using frequencies, ratios, means and medians and the distribution measures were reported using standard deviation and minimum and maximum values. The Kolmogorov-Smirnov test was used to assess whether the total scores of the scale were normally distributed. The Mann-Whitney U test, Kruskal-Wallis analysis and Spearman’s correlation were used because the data were not normally distributed. The significance level was accepted as p<0.05.

## Results

### Study Group

The mean age of the participants (47.2% male, 52.8% female) was 56.6±10.7 years; 74.8% of the participants were under 65 years of age and 25.2% were aged 65 years and over. Sixty percent of the participants were primary school graduates. The distribution of the study group according to selected sociodemographic and medical history characteristics is shown in Table 1.

### Item Discrimination Index and Difficulty Index

Eleven items with an item discrimination index below 0.19 and a difficulty index below 0.29 were removed from the scale. The item discrimination indices ranged from 0.28 to 0.65 and difficulty indices ranged from 33% to 61%.

### Factor Analysis

PCA was done with a varimax rotation. In the factor analysis, the Kaiser-Meyer-Olkin index was 0.760 and the Barlett’s test result was p<0.001. Factor analysis indicated that the single-dimension scale accounted for 26.8% of the total variance. The total correlation values of the items ranged from 24.2% to 42.9%. The factor loadings and reliability values of the GKLQ items are given in Table 2.

### Confirmatory Factor Analysis

**Figure 1:**
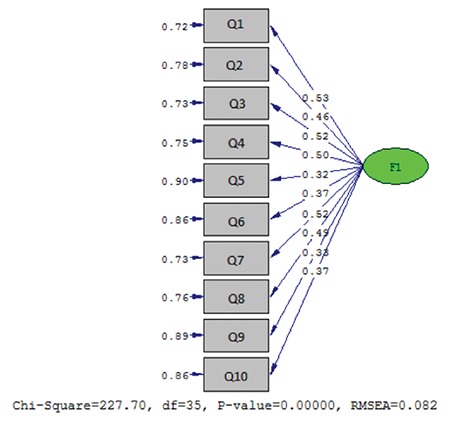
Confirmatory factor analysis diagram for the glaucoma knowledge level questionnaire

After the factors were identified through an exploratory factor analysis, they were tested with CFA to evaluate their consistency with the identified factor constructs. When the fit indices of the model obtained with the CFA were examined, although the c^2^/df value was not below 3, the GFI, CFI and RMSEA values were 0.95, 0.90 and 0.082, respectively, indicating acceptable model fit. In brief, the resulting index of fit values demonstrated good model fit. The fit values of the scale determined in CFA are given in Table 3 and factor loadings pertaining to the model are given in Figure 1.

### Internal Consistency (Reliability)

The internal consistency coefficient (Cronbach’s alpha) of the scale was 0.69. Cronbach’s alpha values with items removed ranged from 0.65 to 0.68.

### Validity

**Figure 2:**
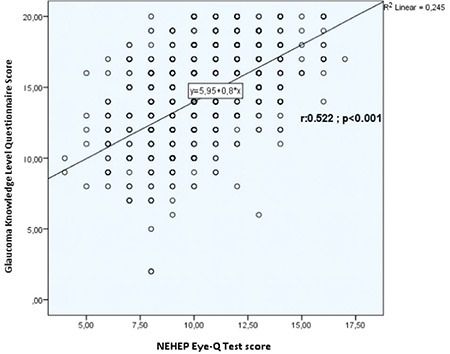
Scatter plot of glaucoma knowledge level questionnaire and National Eye Health Education Program Eye-Q Test scores NEHEP: National Eye Health Education Program

Assessment of validity revealed a positive correlation between the total score of the items in the NEHEP scale and the GKLQ score (r=0.522, p<0.001). The scatter plot of the NEHEP scale and GKLQ scores is presented in Figure 2.

**Figure 3:**
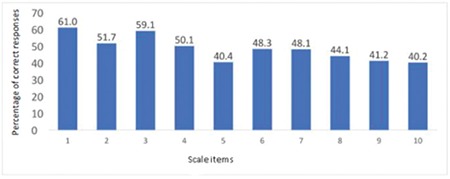
Percentage of participants responding correctly to scale items

In the final version of the scale, the total score possible ranges from 0 to 20 and there is no cut-off score. Higher scores reflect greater knowledge about and awareness of glaucoma. In the study group, the mean (± standard deviation) of the scores obtained from the scale was 13.8±3.3, the median was 14.0 and maximum and minimum scores were 2 and 20. The percentage of correct responses to the GKLQ items varied between 40.2% and 61.0%. The statement with the lowest rate of correct response was “Some medications can cause an increase in eye pressure” and the statement with the highest rate of correct response was “Glaucoma is often the cause of blindness”. The percentages of correct responses to the scale items are presented in Figure 3.

There was no gender-based difference in median GKLQ score. Scores were higher among individuals aged 40-64, those with an education level of high school or higher, those with a good income level, those who had previous eye examinations and those who had previous ocular pressure measurements. Table 4 compares the GKLQ scores of the study group obtained from the GKLQ with their sociodemographic and disease-related characteristics.

## Discussion

The aim of this study was to develop a scale to measure the level of glaucoma knowledge in a community-based sample and to test the validity and reliability of the scale. In order to determine how effectively scale items assess knowledge, they must be evaluated based on item discrimination and difficulty indices. For this scale, item discrimination index values ranged from 0.28 to 0.65 and difficulty index values ranged from 33% to 61%. An item discrimination index of 0.2 or higher is considered acceptable and indicative that the item can distinguish between the unknowledgeable and the knowledgeable.^14^ None of the previously developed glaucoma scales were tested for item discrimination and difficulty indices.

For a reliable scale, the Cronbach’s alpha value should be at least 0.70.^15^ The Cronbach’s alpha value of our scale was 0.69, which was considered adequate. Previously developed glaucoma knowledge scales had lower Cronbach’s alpha values. In fact, although the NEHEP scale is the most widely accepted scale for measuring level of glaucoma knowledge, its Cronbach’s alpha value is 0.59.^16^ Therefore, we believe our scale is reliable. Removal of single items from the scale did not result in a significant increase in the Cronbach’s alpha value, indicating good consistency between the scale items.

CFA was done to ascertain whether the model of the 10-item, one-dimensional GKLQ developed with an EFA was confirmed. The first value to be examined in CFA is the p value. This value indicates the significance of the difference between the expected covariance matrix and the observed covariance matrices (χ^2^). Naturally, a nonsignificant p value is desired. However, it is also normal for the p value to be significant due to a large sample size. In this study, a significant p value was tolerated and alternative fit indices were evaluated.^17^ It is reported that the RMSEA value must be below 0.08 and the GFI and AGFI values must be higher than 0.90 in order for the model fit to be regarded as acceptable.^13^ For this scale, CFA yielded values of 0.082 for RMSEA, 0.95 for GFI and 0.92 for AGFI. These values were evaluated according to fit indices and it was determined that all were at an acceptable level for model fit. Consequently, we consider this evidence that the factor construct resulting from the EFA is strongly confirmed.

We consider the one-dimensional nature of the scale and the small number of items as appropriate for the purpose of the study. There is still no ideal scale for measuring levels of glaucoma knowledge. The NEHEP scale has gained more acceptance compared to other scales. Based on item analyses, three of the items in the NEHEP scale (“Glaucoma is more common among people with glaucoma in their family.”, “The risk of having glaucoma is higher among people over 60 years of age.”, “Glaucoma can be controlled.”) remained in the scale. We believe that the inclusion of items pertaining to the risk factors and treatability of glaucoma in our scale will result in wider acceptance.

Evaluation of GKLQ scores according to sociodemographic characteristics showed that scores were higher among people less than 65 years of age, those living in urban areas, those with education level of high school or higher and those with good income level. These findings are consistent with studies reporting that young age and good socioeconomic and education level are factors that increase knowledge and awareness of glaucoma.^18,19,20^ In addition, the participants in our study group who had previously undergone eye examinations and ocular pressure measurement scored higher on the scale. This supports the reliability of the scale.

## Conclusion

The scale created in this study is not designed to investigate all aspects of glaucoma knowledge. However, the GKLQ is the first scale for determining glaucoma knowledge in Turkey that has been tested for validity and reliability. While previously published tools assessing glaucoma knowledge generally targeted glaucoma patients, the GKLQ is designed as a simple and quick measurement tool that can also be applied to the general population. The reliability of the scale in specific groups needs to be tested and the scale requires further research and development.

## Figures and Tables

**Table 1:**
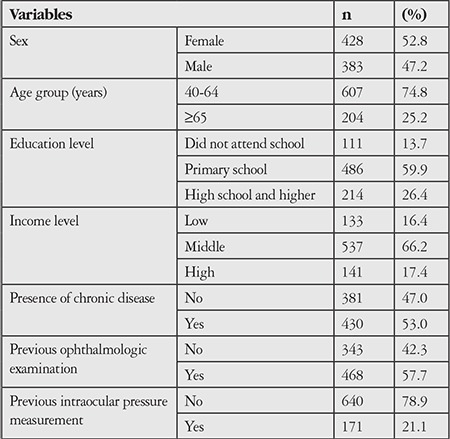
Distribution of the study group according to selected sociodemographic and medical history characteristics

**Table 2:**
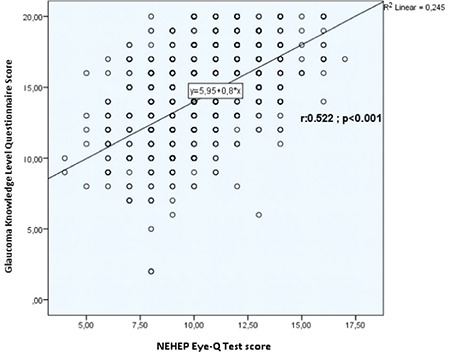
Glaucoma knowledge level questionnaire item factor loadings, corrected item-total correlations and Cronbach’s alpha coefficients if item deleted

**Table 3:**
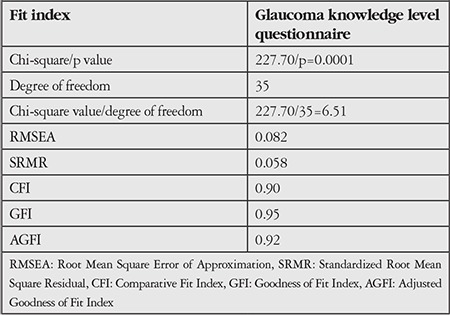
Glaucoma knowledge level questionnaire confirmatory factor analysis fit indices

**Table 4:**
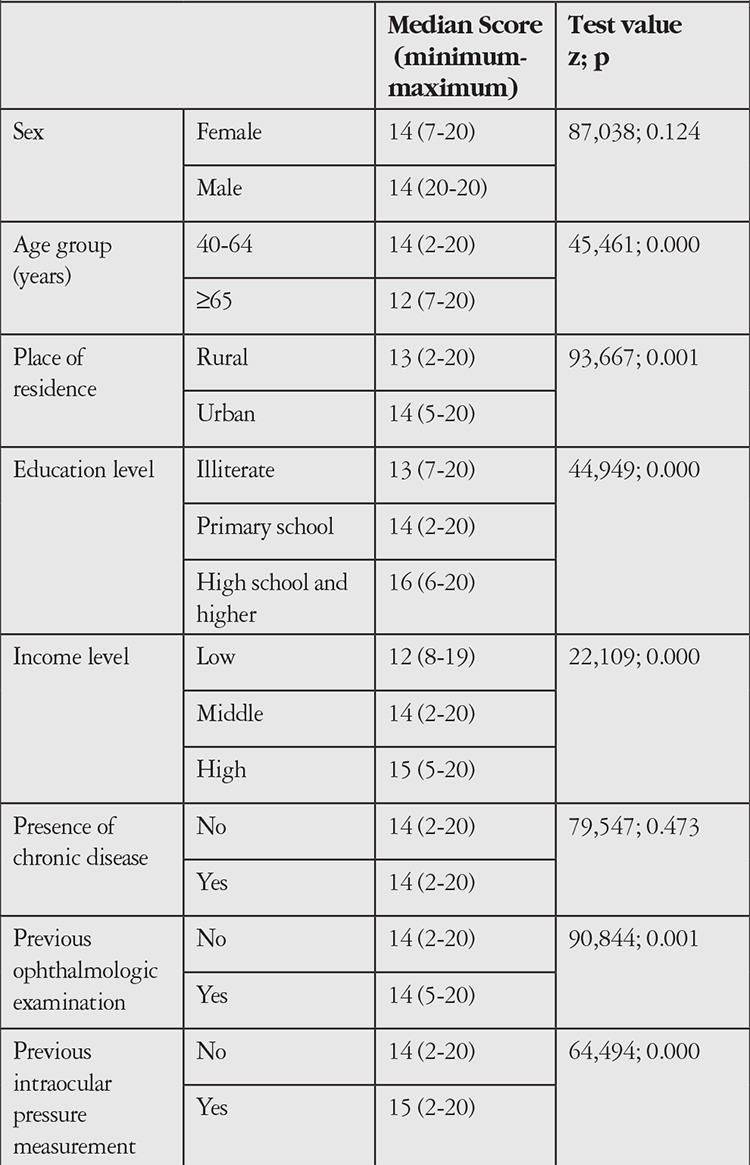
Comparison of median glaucoma knowledge level questionnaire scores of the study group
